# Elbow Precaution

**Published:** 1854-12

**Authors:** 


Elbow Precaution.—A gentleman from Morrisania, had his elbow broken lately, while leaning his arm outside the car on the Harlem railroad. A similar accident befel another gentleman on one of the city cars not long ago. The proper place for the elbow in all vehicles, as at the table and public assemblies, is beside the owner's person; a knowledge and remembrance of this may save some reader of the Journal the price of twenty years subscription. Three-fourths of those who ride in the omnibusses and city cars, sit with the elbows projecting outside: about a year ago, a gentleman had his arm shattered in that position, at the corner of Centre and Canal streets. Another and more fatal form of accident is of almost weekly occurrence, which the delay of three seconds would infallibly prevent. I allude to jumping on and off the city horse cars while in motion, *from the forward platform.* If a man's time is of such immense importance that he must save a single minute at the cost of risking a limb or life, a delay of three seconds will give him a chance of jumping on the rear platform, while the car is passing; if he misses his mark, he has only to get up out of the mud and give his washerwoman an extra shilling or two, while the same "miss" at the forward platform would have thrown him under the wheels and cost him his life, as has just been the case with a city editor, whose very last editorial was said to have been on cautioning his readers against that very practice.

